# FGFR2-Rearranged Biliary Tract Cancer: Biology, Resistance Mechanisms, and Emerging Therapeutic Strategies

**DOI:** 10.3390/cancers18030531

**Published:** 2026-02-06

**Authors:** Xin Xin, Ruoyu Miao

**Affiliations:** 1 St. Joseph’s Hospital, BayCare Health System, Tampa, FL 33607, USA; xin.xin@baycare.org; 2 Department of Hematology and Medical Oncology, Winship Cancer Institute, Emory University, Atlanta, GA 30322, USA

**Keywords:** FGFR2 fusion, cholangiocarcinoma, biliary tract cancer, targeted therapy, resistance mechanisms, futibatinib, pemigatinib, circulating tumor DNA, precision oncology

## Abstract

Fibroblast growth factor receptor 2 (FGFR2) rearrangements represent one of the most actionable molecular alterations in biliary tract cancer, particularly in intrahepatic cholangiocarcinoma (iCCA). Approximately 10–16% of iCCA cases harbor FGFR2 fusions or rearrangements, defining a distinct molecular subtype characterized by sensitivity to FGFR-targeted therapies. Selective FGFR tyrosine kinase inhibitors, including the reversible inhibitor pemigatinib and the irreversible inhibitor futibatinib, have demonstrated clinically meaningful response rates and durable disease control in patients with previously treated FGFR2-altered iCCA, leading to regulatory approvals and the incorporation of FGFR inhibition into contemporary treatment paradigms. However, the development of acquired resistance—most commonly driven by secondary kinase-domain mutations and activation of bypass signaling pathways—remains a major limitation to sustained therapeutic benefit. This review summarizes the biological basis of FGFR2 alterations, highlights current clinical evidence supporting FGFR inhibition, and discusses the evolving landscape of resistance mechanisms. We further examine emerging therapeutic strategies aimed at overcoming resistance, including next-generation FGFR inhibitors and rational combination approaches. In addition, we highlight the growing role of circulating tumor DNA as a noninvasive tool for longitudinal molecular monitoring and treatment guidance. Together, these insights underscore the central role of FGFR2-directed therapy in precision oncology for biliary tract cancer and provide a framework for optimizing and extending targeted treatment in this molecularly defined disease subset.

## Simple Summary

Biliary tract cancer is a rare and aggressive cancer with limited treatment options and poor survival outcomes. In recent years, alterations in fibroblast growth factor receptor 2 (FGFR2) have been identified in a subset of patients, particularly those with intrahepatic cholangiocarcinoma. These genetic changes drive tumor growth but also create an opportunity for targeted treatment. Several drugs that inhibit FGFR2 have shown meaningful clinical benefit; however, most patients eventually develop resistance, leading to disease progression. This review summarizes current knowledge of FGFR2-related cancer biology, available targeted therapies, and the main mechanisms by which resistance develops. We also discuss emerging treatment strategies, including next-generation drugs, combination approaches, and the use of blood-based testing to monitor disease evolution. Improving understanding of these mechanisms may help guide future treatment decisions and improve outcomes for patients with FGFR2-altered biliary tract cancer.

## 1. Introduction

Biliary tract cancers (BTCs) comprise a heterogeneous group of malignancies arising from the epithelium of the intrahepatic and extrahepatic bile ducts as well as the gallbladder. Among these, intrahepatic cholangiocarcinoma (iCCA) has emerged as a subtype with an increasing incidence worldwide and a particularly poor prognosis [1,2]. Despite the introduction of gemcitabine and cisplatin as first-line standard chemotherapy for advanced BTC, the median overall survival (OS) remains below one year [3]. Incorporating immune checkpoint inhibitors (ICIs), such as durvalumab or pembrolizumab, into this regimen has yielded only modest improvements, extending median OS to just over 12 months [4,5,6]. Molecular profiling has significantly advanced the understanding of iCCA, revealing several actionable alterations such as fibroblast growth factor receptor 2 (*FGFR2*) fusions, isocitrate dehydrogenase 1 (*IDH1*) mutations, B-Raf proto-oncogene (*BRAF*) *V600E* mutations, and human epidermal growth factor receptor 2 (*HER2*) amplifications [7,8]. Among these, *FGFR2* rearrangements are particularly notable: they are considered lineage-defining events and predict marked sensitivity to selective FGFR inhibition [9,10,11].

Fibroblast growth factor receptors (FGFRs) are a family of receptor tyrosine kinases that regulate key cellular processes, including cell proliferation, differentiation, survival, and angiogenesis through downstream signaling cascades such as Rat sarcoma(RAS)/mitogen-activated protein kinase (MAPK), phosphoinositide 3-kinase (PI3K)/protein kinase B (AKT), and signal transducer and activator of transcription (STAT) pathways [12]. Aberrant FGFR2 signaling—most commonly arising from gene fusions with partners that induce ligand-independent dimerization—drives oncogenesis by constitutively activating the downstream pathways [13,14]. These findings have led to a therapeutic paradigm shift in BTC, transforming a subset of this historically chemotherapy-refractory disease into a molecularly targetable entity.

The approval of selective FGFR inhibitors, including pemigatinib and futibatinib, has substantially expanded treatment options for patients with *FGFR2* fusion-positive BTC [15,16]. However, secondary resistance typically develops within 12 months of therapy, representing a critical clinical challenge [17,18]. Understanding the molecular mechanisms of resistance and developing strategies to circumvent them are essential for extending therapeutic benefit and improving survival in this molecularly defined subset of BTC.

## 2. Molecular Biology and Clinical Significance of FGFR2 Alterations

### 2.1. Structure and Function of FGFR2

The *FGFR2* gene, located on chromosome 10q26, encodes a transmembrane receptor tyrosine kinase comprising three extracellular immunoglobulin-like domains, a single transmembrane helix, and an intracellular tyrosine kinase domain [12]. FGFR2 consists of three extracellular immunoglobulin-like domains (D1–D3), an acidic box that modulates ligand binding, a single-pass transmembrane helix, and an intracellular split tyrosine kinase domain. Alternative splicing of the D3 domain generates the IIIb and IIIc isoforms, which display distinct ligand-binding specificities and tissue-restricted expression patterns, contributing to epithelial–mesenchymal signaling diversity [19,20].

**Figure 1:**
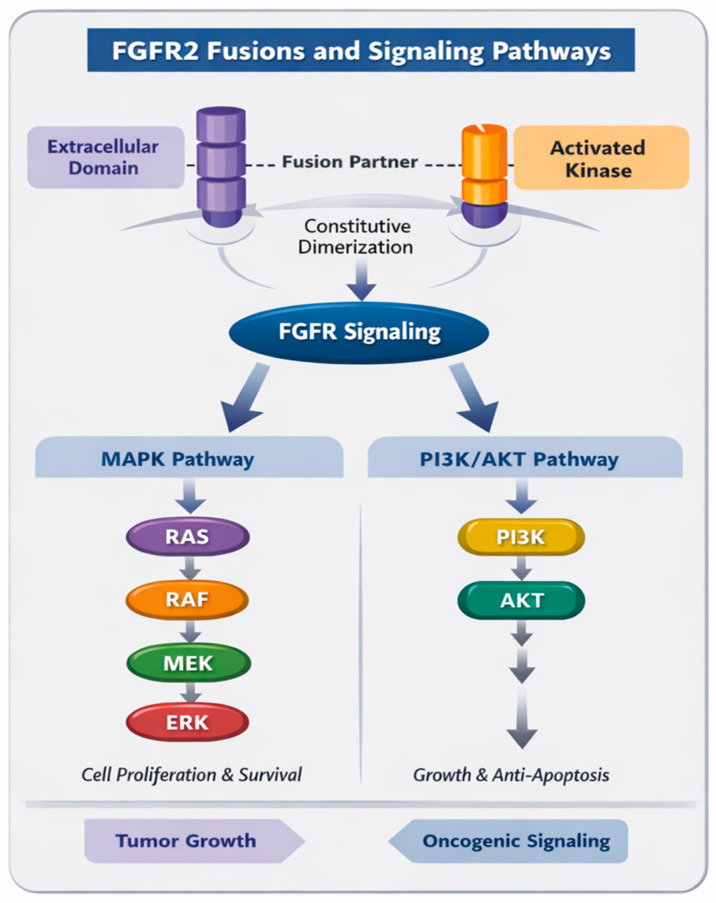
Fibroblast growth factor receptor 2 (*FGFR2*) fusion biology and oncogenic signaling pathways in intrahepatic cholangiocarcinoma. *FGFR2* rearrangements generate fusion proteins that retain the intracellular kinase domain and acquire oligomerization motifs from partner genes, leading to ligand-independent receptor dimerization and constitutive activation. Activated FGFR2 fusion proteins engage multiple downstream signaling cascades, most prominently the mitogen-activated protein kinase (MAPK) (Rat sarcoma [RAS]–rapidly accelerated fibrosarcoma kinase [RAF]–mitogen-activated protein kinase [MEK]–extracellular signal-regulated kinase [ERK]) and, phosphoinositide 3-kinase (PI3K)–protein kinase B (AKT) pathways, promoting tumor cell proliferation, survival, and resistance to apoptosis. Aberrant FGFR2 signaling also contributes to tumor growth and oncogenic dependency, establishing FGFR2 as a validated therapeutic target in *FGFR2*-rearranged intrahepatic cholangiocarcinoma.

Physiological activation of FGFR2 requires binding of fibroblast growth factors (FGFs) in the presence of heparan sulfate proteoglycans, which stabilize ligand-receptor complexes and promote receptor dimerization. This induces trans-autophosphorylation of intracellular tyrosine residues, allowing recruitment of adaptor proteins such as fibroblast growth factor receptor substrate 2 (FRS2) and growth factor receptor-bound protein 2 (GRB2) [19,21]. Activated FGFR2 engages multiple downstream signaling pathways including RAS/rapidly accelerated fibrosarcoma kinase (RAF)/mitogen-activated protein kinase (MEK)/extracellular signal-regulated kinase (ERK), PI3K/AKT/mechanistic target of rapamycin (mTOR), phospholipase C γ (PLCγ), and Janus kinase (JAK)/STAT signaling, which collectively regulate cellular proliferation, survival, differentiation, migration, and angiogenesis [21,22]. Oncogenic activation of FGFR2 can arise through multiple mechanisms, such as gene amplification, activating mutations, or chromosomal rearrangements resulting in *FGFR2* fusions, the latter representing the dominant oncogenic mechanism in BTC (Figure 1).

### 2.2. Prevalence and Oncogenic Drivers in BTC

Large-scale genomic profiling efforts have established that *FGFR2* rearrangements are a defining molecular event in a substantial subset of iCCA, occurring in approximately 10–16% of cases across Western and Asian cohorts, while remaining rare in extrahepatic cholangiocarcinoma (eCCA) and gallbladder cancer (GBC) [23,24]. Among recurrent genomic alterations in iCCA, *IDH1* mutations are observed at a similar frequency (approximately 10–15%), whereas alterations such as *BRAF V600E* mutations, neurotrophic tyrosine receptor kinase (*NTRK*) fusions, and anaplastic lymphoma kinase (*ALK*)/c-ros oncogene 1 (*ROS1*) rearrangements are distinctly uncommon. In contrast, erb-b2 receptor tyrosine kinase 2 (*ERBB2* or *HER2*) amplification is infrequent in iCCA but more prevalent in eCCA and GBC, underscoring the anatomic specificity of actionable drivers within biliary tract cancer (Table 1) [9,10,25]. Such differences likely reflect heterogeneity in etiologic risk factors and underlying liver disease.

Clinically, *FGFR2* fusion-positive iCCA has been associated in several retrospective series with younger age at diagnosis, female predominance, and, in some cohorts, a comparatively less aggressive clinical course [26]. However, these observations may be influenced by selection effects, referral patterns, and treatment-related factors, and should therefore be interpreted cautiously. From a therapeutic standpoint, *FGFR2* fusions uniquely define a molecularly enriched disease subset supported by multiple selective FGFR inhibitors demonstrating reproducible objective responses and durable disease control across independent phase II trials [5,10,27]. This depth of clinical validation, together with the clear lineage restriction of *FGFR2* rearrangements to iCCA, distinguishes FGFR2 from other targets and underpins its current role as one of the most therapeutically actionable drivers in BTC.

More than 100 distinct fusion partners have been identified to date, including *BICC1*, *PPHLN1*, *KIAA1598* (*SHROOM3*), and *TACC3*, most of which contribute oligomerization domains that drive constitutive receptor dimerization. These rearrangements preserve the FGFR2 kinase domain while removing regulatory regions, resulting in ligand-independent activation of downstream oncogenic signaling pathways [13,14,28]. Available evidence suggests that many *FGFR2* fusions share common oncogenic properties driven by constitutive kinase activation, regardless of fusion partner; however, biological heterogeneity likely exists, and the extent to which specific fusion partners influence signaling output, tumor biology, or therapeutic sensitivity remains an active area of investigation.

### 2.3. Downstream Signaling and Biological Effects

Constitutive activation of FGFR2 fusion proteins leads to persistent, ligand-independent stimulation of downstream oncogenic signaling, most prominently the MAPK (RAS/RAF/MEK/ERK) and PI3K/AKT/mTOR axes, thereby promoting sustained cellular proliferation, survival, and resistance to apoptosis (Figure 1) [20,29,30]. Beyond tumor cell-intrinsic effects, aberrant FGFR2 activation may further modulate the tumor microenvironment (TME) by inducing pro-angiogenic factors, including VEGF, and engaging in paracrine crosstalk with endothelial cells and cancer-associated fibroblasts, thereby facilitating stromal remodeling, epithelial–mesenchymal transition (EMT), and invasive behavior [30,31]. Integrative genomic and transcriptomic analyses have demonstrated that *FGFR2* fusion-positive iCCA constitutes a distinct molecular lineage characterized by low tumor mutation burden and an immune-cold phenotype with limited cytotoxic T-cell infiltration and low expression of immune checkpoint molecules [32,33], which may partly explain the limited clinical activity of ICIs observed in this subtype.

### 2.4. Clinical Significance

*FGFR2* fusions represent a validated predictive biomarker for response to FGFR-targeted therapies in iCCA. Across multiple phase II studies, selective FGFR inhibitors, including pemigatinib, infigratinib, and futibatinib, have demonstrated reproducible antitumor activity in patients with *FGFR2* fusion-positive iCCA, with objective response rates (ORR) of approximately 20–40% and median progression-free survival (PFS) of 6–9 months. These outcomes compare favorably with those historically reported for cytotoxic chemotherapy in advanced biliary tract cancer. However, it is important to emphasize that these data derive predominantly from single-arm trials and basket study subsets, and that any comparisons with historical chemotherapy cohorts are inherently indirect. Differences in patient selection, prior lines of therapy, molecular enrichment, and assessment methodologies limit cross-trial interpretability, and preclude definitive conclusions regarding relative efficacy [15,16,34,35]. Although responses to FGFR inhibition can be durable, most patients ultimately develop acquired resistance. Nevertheless, the consistent clinical benefit observed across multiple agents has validated FGFR2 as a therapeutic target and transformed the treatment paradigm for this molecularly defined subgroup. Accordingly, comprehensive genomic profiling should therefore be routinely performed for all patients with advanced iCCA to identify *FGFR2* fusions and other actionable alterations and inform targeted therapy selection [36].

## 3. Clinical Development of FGFR Inhibitors in Biliary Tract Cancer

### 3.1. Overview of FGFR-Targeted Agents

Therapeutic development of FGFR inhibitors has largely centered on small-molecule tyrosine kinase inhibitors (TKIs) that target the ATP-binding pocket of the FGFR kinase domain. These agents include reversible, ATP-competitive inhibitors, such as pemigatinib, infigratinib, derazantinib, and erdafitinib, as well as irreversible covalent inhibitors exemplified by futibatinib, which forms a covalent bond with a conserved cysteine residue within the FGFR kinase domain. Although these compounds differ in their biochemical potency and selectivity profiles across FGFR1-4, they share a common mechanism of suppressing aberrant FGFR-driven signaling through inhibition of downstream pathways. Across multiple phase I-II clinical trials, these agents have demonstrated reproducible and clinically meaningful antitumor activity in pretreated patients with *FGFR2* fusion-positive cholangiocarcinoma (CCA). The consistent clinical activity of these agents, together with the relative lack of activity in tumors lacking *FGFR2* rearrangements, has established *FGFR2* fusions as validated oncogenic drivers and therapeutic targets [15,16,27,34,37,38,39,40,41]. Table 2 summarizes the approved and investigational FGFR inhibitors for BTC.

### 3.2. Key Clinical Trials

FIGHT-202 was a pivotal phase II trial evaluating the selective FGFR1-3 inhibitor pemigatinib in patients with previously treated, locally advanced or metastatic CCA [15,38]. Among 108 patients with *FGFR2* fusions or rearrangements, the ORR was 37.0% (95% confidence interval [CI]: 27.9–46.9), with a median duration of response (DOR) of 9.1 months (95% CI: 6.0–14.5). No objective responses were observed among patients with other *FGF*/*FGFR* alterations or without *FGF*/*FGFR* alterations. Median PFS was 7.0 months (95% CI: 6.1–10.5) while median OS was 17.5 months (95% CI: 14.4–22.9) among patients with *FGFR2* fusions or rearrangements. The most common treatment emergent adverse events (TEAEs) were hyperphosphatemia, alopecia, diarrhea, stomatitis, dysgeusia, and fatigue [38]. The randomized phase III FIGHT-302 trial (NCT03656536) was designed to compare pemigatinib with gemcitabine plus cisplatin in the first-line treatment of advanced CCA with *FGFR2* rearrangements [42]. According to publicly available ClinicalTrials.gov records, the study is listed as discontinued, with the most recent update posted on 24 August 2025, citing enrollment challenges in the context of an evolving first-line treatment landscape.

A phase II single-arm study of the selective FGFR1-3 inhibitor infigratinib in patients with previously treated CCA harboring *FGFR2* fusions or rearrangements demonstrated an ORR of 23.1% (95% CI: 15.6–32.2), a median PFS of 7.3 months (95% CI: 5.6–7.6), and a median OS of 12.2 months (95% CI: 10.7–14.9) [34]. Despite the promising clinical activity, the subsequent development program was later discontinued, and the U.S. Food and Drug Administration (FDA) approval for infigratinib in this indication was voluntarily withdrawn following a strategic and commercial review by the sponsor.

Similarly, the multikinase FGFR1-3 inhibitor derazantinib (ARQ 087) demonstrated moderate clinical activity in patients with *FGFR2* fusion- or rearrangement-positive iCCA in the phase II FIDES-01 study, with an ORR of 21.4% (95% CI: 13.9–30.5) and a median DOR of 6.4 months (95% CI: 3.9–9.2). Median PFS and OS were 7.8 months (95% CI: 5.5–8.2) and 15.5 months (95% CI: 11.8–21.9), respectively, with a manageable toxicity profile [27,39]. Although subsequent evaluation in metastatic urothelial cancer (FIDES-02) did not demonstrate sufficient clinical benefit [43], and development was ultimately discontinued following strategic reprioritization, early results in iCCA supported FGFR2 as a biologically relevant therapeutic target.

FOENIX-CCA2 was a single-arm, global phase II trial evaluating futibatinib (TAS-120), an irreversible covalent FGFR1-4 inhibitor, in patients with previously treated iCCA harboring *FGFR2* fusions or rearrangements [16,40]. Among 103 evaluable patients, the ORR was 42% (95% CI: 32–52), with a median DOR of 9.7 months (95% CI: 7.6–17.0). Median PFS was 9.0 months (95% CI: 6.9–13.1), and median OS was 21.7 months (95% CI: 14.5-not reached [NR]). The most common side effects were hyperphosphatemia, alopecia, and dry mouth [40]. Notably, futibatinib also demonstrated activity in a subset of patients previously treated with reversible FGFR inhibitors, supporting its ability to overcome certain acquired resistance mutations [44,45]. FOENIX-CCA3 (NCT04093362) was a randomized phase III study evaluating futibatinib in the first line setting for patients with *FGFR2*-rearranged iCCA. Based on ClinicalTrials.gov (accessed on 28 January 2026) registry information, the trial is currently listed as discontinued, with the most recent update posted on 7 February 2025, noting recruitment challenges after changes in the standard-of-care landscape. Subsequent clinical development has focused on dose and exposure optimization, including the ongoing FOENIX-CCA4 study (NCT05727176).

The selective pan-FGFR inhibitor erdafitinib was evaluated in the phase II RAGNAR basket trial in previously treated patients with *FGFR1-4*-altered advanced non-urothelial solid tumors [46]. In an exploratory subgroup of 35 patients with CCA, preliminary results showed an ORR of 60.0% (95% CI: 42.1–76.1) with a median DOR of 5.6 months (95% CI: 2.8–8.3). Responses were observed in patients harboring *FGFR* fusions and activating mutations. Disease control was achieved in nearly all treated patients. Median PFS and OS were 8.4 months (95% CI: 5.5–9.7) and 18.7 months (95% CI: 8.9–not evaluable [NE]), respectively. Safety profile was similar to that of other FGFR inhibitors, with the most common TEAEs being hyperphosphatemia, diarrhea, and stomatitis [41]. These findings suggest promising activity of erdafitinib in selected *FGFR*-altered CCA.

Collectively, these data establish FGFR inhibition as a clinically meaningful therapeutic strategy in molecularly selected BTCs and support incorporation of *FGFR* testing into routine diagnostic workflows.

## 4. Mechanisms of Resistance to FGFR Inhibition

### 4.1. Overview

**Figure 2:**
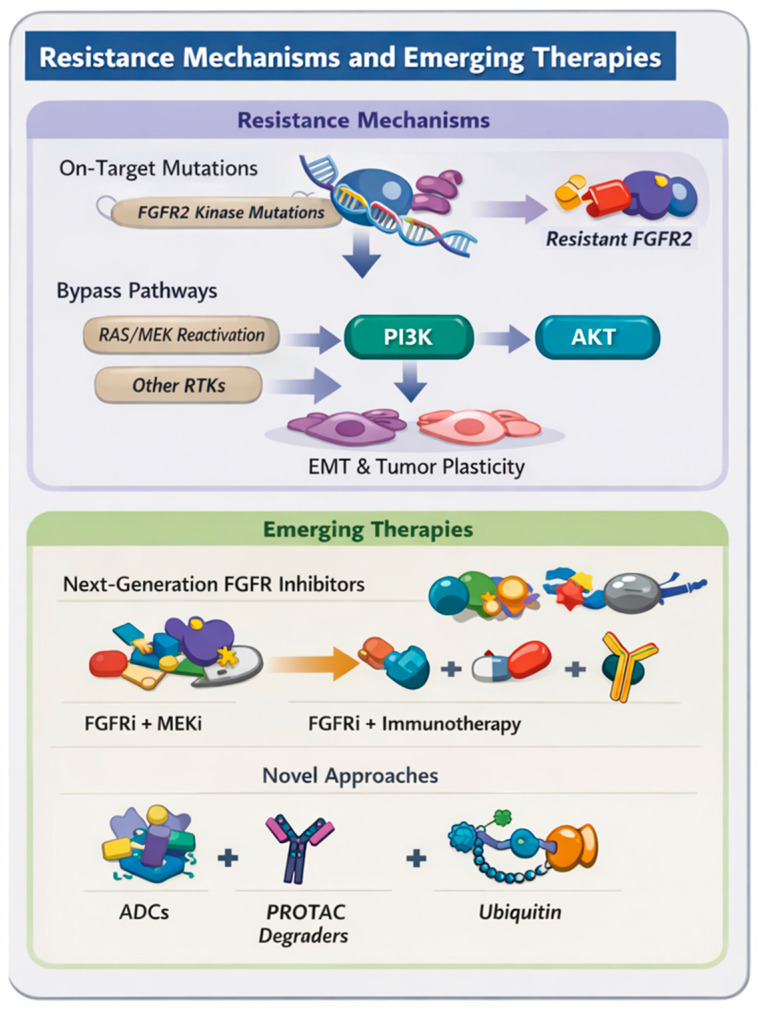
Mechanisms of resistance to fibroblast growth factor receptor (FGFR) inhibition and emerging therapeutic strategies in *FGFR2*-rearranged intrahepatic cholangiocarcinoma. Acquired resistance to FGFR inhibitors arises through both on-target and off-target mechanisms. Upper panel (On-Target Mutations): On-target resistance is most commonly driven by secondary *FGFR2* kinase-domain mutations, including gatekeeper and molecular-brake alterations, which impair inhibitor binding and often emerge in a polyclonal pattern. The DNA double helix represents genetic alterations acquired during treatment. The FGFR protein structure denotes the FGFR2 receptor. The pill icon represents FGFR-targeted therapy. The modified FGFR receptor signifies a drug-resistant FGFR2 protein. Upper panel (Bypass Pathways): Off-target mechanisms include activation of bypass signaling pathways such as mitogen-activated protein kinase (MAPK) and phosphoinositide 3-kinase (PI3K)/protein kinase B (AKT) through alternative receptor tyrosine kinases (RTKs) or downstream mutations, as well as phenotypic plasticity and epithelial–mesenchymal transition. Lower panel: Emerging therapeutic strategies aimed at overcoming resistance include next-generation and covalent FGFR inhibitors, rational combination approaches targeting bypass pathways, and non-tyrosine kinase inhibitor modalities such as antibody-drug conjugates and proteolysis-targeting chimera (PROTAC) degraders.

Despite initial responses, virtually all patients treated with FGFR TKIs eventually experienced disease progression, reflecting the development of acquired resistance. The median duration of response across clinical trials is approximately 6–9 months, with some variability by agent [27,34,38,40,41]. Molecular analyses of post-progression tumor biopsies and circulating tumor DNA (ctDNA) have delineated two principal categories of resistance mechanisms: on-target mutations within the FGFR2 kinase domain and off-target or bypass pathway alterations that restore downstream signaling independently of FGFR2 (Table 3 and Figure 2) [17,18,47,48].

### 4.2. On-Target Resistance Mechanisms

The most frequent mechanism of acquired resistance to FGFR inhibition in *FGFR2* fusion-positive CCA involves the emergence of secondary mutations within the FGFR2 kinase domain that impair inhibitor binding affinity. These alterations typically arise under selective pressure from ATP-competitive FGFR inhibitors and cluster within structurally and functionally critical regions of the kinase domain, such as the ATP-binding cleft, the gatekeeper residue, and the molecular brake motif that normally maintains kinase autoinhibition [17,49].

Recurrent gatekeeper substitutions at valine 565 (V565F/L/I) increase steric hindrance within the ATP-binding pocket and reduce the binding affinity of reversible FGFR inhibitors [17,49,50]. In parallel, molecular-brake mutations involving *N550D/K/H*, *E566A*, or *K659M* destabilize the inactive conformation of FGFR2, enhance constitutive receptor phosphorylation, and sustain downstream MAPK and PI3K signaling despite continued drug exposure [49,50,51]. Importantly, distinct resistance mutations confer heterogeneous effects on inhibitor sensitivity, highlighting the structural diversity of on-target resistance mechanisms [17].

Acquired resistance is further complicated by its frequently polyclonal nature. Post-progression analyses have demonstrated the coexistence of multiple *FGFR2* kinase-domain mutations within individual patients, sometimes varying across metastatic sites or evolving dynamically over time [48,49,52]. This spatial and temporal heterogeneity reflects convergent evolutionary pressure and underscores the biological complexity of resistance development in FGFR2-driven disease.

### 4.3. Off-Target and Bypass Signaling Mechanisms

Not all resistance to FGFR inhibition is mediated by structural alterations of FGFR2. A subset of tumors develops off-target resistance mechanisms that bypass FGFR dependency through activation of alternative growth and survival pathways capable of re-engaging downstream signaling [53,54]. In this context, tumor cells maintain proliferative signaling despite sustained pharmacologic FGFR blockade, representing a mechanistically distinct form of resistance from kinase-domain mutation-driven escape.

The most commonly implicated bypass mechanism involves reactivation of the MAPK pathway, driven by acquired alterations in RAS or BRAF or by upregulation of alternative receptor tyrosine kinases such as MET, epidermal growth factor receptor (EGFR), or ERBB2 [55,56,57]. These changes restore ERK phosphorylation independently of FGFR2 activity and support continued tumor growth.

In addition to genomic alterations, tumors may adopt non-genetic adaptive programs. Transcriptional and epigenetic reprogramming, including epithelial–mesenchymal transition (EMT), has been observed in resistant models and clinical samples [58,59]. EMT is characterized by reduced epithelial marker expression and increased levels of mesenchymal regulators such as TWIST1, SNAIL, and ZEB1, promoting phenotypic plasticity and diminished dependence on FGFR2 signaling [60].

### 4.4. Role of Circulating Tumor DNA in Resistance Detection

Serial monitoring of circulating tumor DNA (ctDNA) has substantially enhanced the ability to characterize resistance evolution in patients receiving FGFR-targeted therapy. Multiple studies specifically in *FGFR2*-rearranged iCCA have demonstrated that ctDNA profiling can detect emergent *FGFR2* kinase-domain mutations prior to radiographic progression, often providing clinically meaningful lead time and revealing marked intrapatient heterogeneity of resistance mechanisms [61].

In addition, important insights have come from basket trial populations that included, but were not limited to, CCA. In the FIGHT-207 basket study of pemigatinib in advanced solid tumors harboring *FGFR1*-*3* alterations, which enrolled a subset of patients with CCA, longitudinal ctDNA analysis demonstrated that secondary *FGFR2* mutations emerged almost exclusively in patients who initially derived clinical benefit, supporting the role of selective therapeutic pressure in shaping resistance [1].

Together, these CCA-specific cohorts and basket trial analyses establish ctDNA as a powerful, minimally invasive tool to capture the temporal and polyclonal nature of resistance to FGFR inhibition, although the relative sensitivity of plasma-based assays and the generalizability of findings across tumor types must be carefully considered.

## 5. Strategies to Overcome Resistance and Future Directions

### 5.1. Sequential and Next-Generation FGFR Inhibitors

Sequential use of FGFR inhibitors, often informed by emerging resistance mechanisms, has become an important exploratory therapeutic strategy in *FGFR2*-rearranged iCCA. Patients progressing on reversible ATP-competitive inhibitors who develop on-target kinase domain mutations may derive benefit from switching to irreversible inhibitors. Futibatinib, which forms a covalent bond with a conserved cysteine residue in the FGFR2 kinase domain, retains activity against multiple secondary *FGFR2* mutations, although incomplete mutation coverage remains a challenge [16,62].

Beyond currently approved agents, ongoing structure-guided drug development and medicinal chemistry efforts aim to refine FGFR inhibition and address the molecular heterogeneity of resistance in FGFR2-driven cancers by leveraging multiple complementary strategies. One active direction is the design of next-generation reversible inhibitors with structure-optimized ATP-binding pockets and hinge interactions to better accommodate steric hindrance introduced by gatekeeper and molecular-brake mutations such as V565 and N550 variants [63,64,65]. In parallel, investigational covalent FGFR inhibitors are being optimized to improve engagement of conserved residues within the kinase domain and maintain potency against a broader array of resistant alleles while balancing selectivity and toxicity [66,67,68]. Recent structure-based scaffold redesign has yielded selective FGFR2/3 inhibitors with improved biochemical profiles against wild-type and mutant kinases, underscoring how rational scaffold modification can enhance both selectivity and resistance coverage [69]. Several next-generation inhibitors and allele-specific compounds are currently in early clinical development [70].

Tinengotinib (TT-00420) is a spectrum-selective, multikinase inhibitor with potent activity against FGFR1-3 along with Aurora A/B, VEGFR, JAK1/2, and CSF1R that has demonstrated clinical activity in patients with *FGFR*-altered CCA, including those previously treated with FGFR inhibitors [71]. In a phase II study (NCT04919642), tinengotinib showed objective responses across FGFR alteration subgroups, albeit with limited sample sizes. ORR was 6.3% (95% CI: 0.2–30.2) in patients with *FGFR2* fusions and primary FGFR inhibitor resistance, 30.0% (95% CI: 6.7–65.3) in patients with *FGFR2* fusions and acquired resistance, 23.1% (95% CI: 5.0–53.8) in other FGFR alterations, but 0% in FGFR wild type. Exploratory analyses suggested improved outcomes in FGFR inhibitor-naïve patients compared with those previously exposed, underscoring the continued impact of prior FGFR therapy on subsequent treatment efficacy [72].

More selective FGFR2 inhibitors have been developed to enhance on-target potency while minimizing off-target toxicity. Lirafugratinib (RLY-4008), a highly FGFR2-selective, covalent inhibitor, exhibits robust preclinical activity against common resistance mutations and avoids FGFR1- and FGFR4-mediated toxicities [67]. Early clinical data from the phase I/II ReFocus study (NCT04526106) demonstrated substantial activity in FGFR inhibitor-naïve *FGFR2* fusion- or rearrangement-positive CCA [73], with more modest responses in patients previously treated with FGFR inhibitors, consistent with a resistance-driven disease biology [74]. Tasurgratinib (E7090), an irreversible, ATP competitive FGFR1-3 inhibitor, has also shown promising early activity in *FGFR2*-rearranged CCA in a phase I study (NCT02275910), although data remain preliminary [75]. SURF201 (NCT06160752) is investigating TYRA-200, an oral covalent FGFR1/2/3 inhibitor, in patients with advanced iCCA and other solid tumors with primary activating alterations in *FGFR2* and on-target acquired *FGFR2* resistance mutations [68]. Collectively, these next-generation agents illustrate evolving strategies to extend clinical benefit through improved kinase selectivity and broader coverage of resistance mutations.

### 5.2. Combination Strategies Targeting Bypass Pathways

Given the frequent activation of alternative signaling routes following FGFR inhibition, rational combination therapies have emerged primarily from preclinical and early translational studies as an attractive strategy to delay or overcome acquired resistance. Combination approaches have focused on simultaneously targeting FGFR2 and key downstream or parallel signaling nodes that sustain tumor growth despite receptor blockade.

In preclinical models of *FGFR2* fusion-positive CCA, combinations of FGFR inhibitors with agents targeting the MAPK pathway or selected receptor tyrosine kinases have demonstrated synergistic antitumor activity, including enhanced growth suppression and increased apoptotic responses in vitro and in vivo [53,57]. These studies provide proof-of-concept that dual-pathway inhibition may delay the expansion of resistant subclones. However, clinical validation of these approaches remains limited, and their safety, tolerability, and efficacy require prospective evaluation in early-phase clinical trials.

In parallel, combination strategies incorporating inhibitors of the PI3K/AKT/mTOR axis have been investigated in preclinical FGFR-driven CCA models. Co-targeting FGFR and PI3K–mTOR signaling has been shown to restore sensitivity to FGFR inhibition and enhance cytotoxic responses, including induction of autophagic cell death in resistant models [54,76]. While these findings highlight the therapeutic potential of multi-pathway suppression, PI3K–mTOR-based combination approaches remain investigational and should currently be pursued within carefully designed clinical trials.

### 5.3. Role of Antibody-Based and Non-TKI Strategies

Beyond small-molecule kinase inhibitors, alternative therapeutic strategies that remain largely preclinical are being explored to target FGFR2 signaling while bypassing resistance driven by kinase-domain mutations. Beyond classical inhibition, proteolysis-targeting chimera (PROTAC)-based degraders directed against the FGFR family are emerging as a promising investigational non-occupancy-based therapeutic modality [64]. Early FGFR-directed degraders have demonstrated potent depletion of FGFR2 and antiproliferative activity in FGFR-dependent models [77,78], establishing the feasibility of targeted FGFR degradation.

Although FGFR2-selective degraders and constructs engineered to address gatekeeper mutations remain in early preclinical development, the PROTAC approach offers a mechanistically distinct strategy to eliminate both wild-type and mutant FGFR2 proteins rather than merely inhibit their kinase activity [64,79]. As such, PROTAC-based FGFR targeting represents a compelling translational concept, though substantial optimization and clinical evaluation will be required before applicability in biliary tract cancer can be established.

Additional innovations include antibody-based approaches that target the extracellular domain of FGFR2, enabling ligand blockade and receptor inhibition independent of ATP-binding site conformation and potentially retaining activity despite certain kinase-domain mutations. Antibody-based FGFR2-targeted strategies have shown proof-of-concept activity in preclinical studies, but clinical development has thus far been limited, and no antibody-based FGFR2 therapies are currently approved for biliary tract cancer.

The earliest clinical agent, aprutumab ixadotin (BAY1187982), an FGFR2-directed antibody-drug conjugate (ADC), demonstrated selective cytotoxicity in FGFR2-expressing preclinical models and early signs of antitumor activity; however, dose-limiting ocular and hematologic toxicities, together with limited clinical efficacy, led to early termination of its first-in-human study in advanced solid tumors [80]. Nonetheless, its development established proof of concept for FGFR2-targeted biologics as an alternative to kinase inhibition. Parallel efforts are now focused on engineering next-generation ADCs with improved antibody backbones and refined linker-payload designs to achieve greater tumor selectivity while minimizing systemic toxicity [81,82]. Biparatopic antibodies that bind multiple non-overlapping extracellular FGFR2 epitopes demonstrate enhanced receptor blockade, internalization, and antitumor activity in preclinical *FGFR2* fusion-driven CCA models, including activity against kinase-mutant variants, supporting their potential as innovative therapeutics [83]. Additional modalities, including FGFR-targeted bispecific antibodies, FGF-ligand-trap fusion proteins, and FGFR-directed CAR-T constructs, are being explored across FGFR-altered solid tumors, but data specific to *FGFR2*-fusion or *FGFR2*-mutant CCA are still limited [84,85,86]. These emerging biologic strategies offer mechanistically orthogonal approaches to ATP-competitive inhibition and may ultimately provide therapeutic options for patients with TKI-refractory FGFR2-driven disease once further preclinical and clinical evaluation is completed (Figure 2).

### 5.4. Integration of ctDNA-Guided Precision Oncology

The integration of ctDNA profiling into clinical workflows represents a promising advance for patients with *FGFR2*-rearranged iCCA. In CCA-focused studies, serial plasma analysis has enabled non-invasive detection of emergent on-target resistance mutations, delineation of polyclonal evolution, and dynamic monitoring of molecular response during FGFR inhibitor therapy [1,61].

Insights from larger basket trials that included CCA, such as FIGHT-207, further demonstrate the feasibility of longitudinal ctDNA monitoring across *FGFR*-altered solid tumors and support its role in identifying resistance mechanisms that may inform therapeutic sequencing or clinical trial selection. However, because such studies enroll heterogeneous tumor types, extrapolation to CCA should be performed cautiously and interpreted in the context of disease-specific datasets [87].

As ctDNA technologies mature, prospective studies focused specifically on *FGFR2*-rearranged CCA will be critical to define standardized testing intervals, analytic thresholds, and actionable resistance frameworks [88].

### 5.5. Clinical Implications and Treatment Sequencing Considerations

In current clinical practice, FGFR inhibitors are typically employed in patients with advanced *FGFR2*-rearranged iHCC following progression on first-line chemotherapy, consistent with regulatory approvals and guideline recommendations. Selection among available FGFR inhibitors is influenced by prior FGFR inhibitor exposure, toxicity profiles, and the presence of known resistance mutations.

Repeating molecular assessment at disease progression is increasingly important. Tissue biopsy remains the gold standard for comprehensive genomic characterization; however, serial ctDNA analysis offers a minimally invasive alternative that can capture emerging resistance mutations and polyclonal evolution in real time. In practice, ctDNA profiling is particularly useful when tissue acquisition is not feasible or when rapid therapeutic decision-making is required, while tissue biopsy may be preferred when histologic transformation or non-genomic resistance is suspected.

Next-generation FGFR inhibitors and rational combination strategies are most appropriately considered in the context of clinical trials, especially for patients with acquired resistance to first-generation FGFR inhibitors. As prospective data mature, integrating resistance-guided sequencing strategies may further refine personalized treatment approaches for *FGFR2*-rearranged disease.

### 5.6. Adaptive Clinical Trial Designs

Beyond current clinical practice, adaptive platform trial designs that allocate patients to treatment arms based on molecular resistance profiles represent an efficient and increasingly attractive framework for evaluating emerging therapies in precision oncology. Adaptive platform trials that allocate patients to treatment arms based on molecular resistance profiles represent an efficient and increasingly attractive framework for evaluating emerging therapies in precision oncology. Such designs facilitate the rapid assessment of combination regimens or allele-specific inhibitors in small, molecularly defined cohorts [89]. Integrating real-time ctDNA monitoring within these frameworks could enable earlier identification of molecular progression, support adaptive treatment modification, improve response durability, and generate longitudinal data to refine molecular response endpoints, although widespread clinical implementation remains investigational [90].

## 6. Conclusions

The discovery of *FGFR2* rearrangements as a defining oncogenic driver in intrahepatic cholangiocarcinoma has transformed the therapeutic landscape of biliary tract cancer. The development of selective FGFR inhibitors such as pemigatinib and futibatinib represents a major advance in precision oncology for this rare but aggressive malignancy, supported by consistent clinical activity across multiple phase II trials and resulting in regulatory approvals. FGFR2-directed therapy is now an established molecularly guided treatment option for patients with *FGFR2*-rearranged disease. Nevertheless, acquired resistance—driven by heterogeneous kinase-domain mutations and bypass signaling—remains a formidable obstacle to long-term disease control.

Ongoing translational and clinical research is focused on elucidating the molecular basis of resistance, optimizing sequencing strategies, and developing next-generation or combination regimens to extend the benefit of FGFR inhibition. Incorporating serial ctDNA profiling as an emerging clinical tool may enable dynamic, mutation-guided therapy selection and earlier identification of resistance. Ultimately, multidisciplinary collaboration among clinicians, translational scientists, and trial investigators will be essential to fully realize and extend the potential of FGFR2-directed precision therapy in biliary tract cancer.

## Figures and Tables

**Table 1:** Major actionable molecular targets in biliary tract cancer.

Molecular Alteration (Biomarker)	Predominant BTC Subtype(s)	Approx. Prevalence (by Subtype)	Representative Targeted Therapy Options	Highest Level of Clinical Evidence
*FGFR2* fusion/rearrangement	iCCA	iCCA: ~10–15%|eCCA/GBC: rare	Pemigatinib, futibatinib	Phase II trials
*IDH1* mutation	iCCA	iCCA: up to ~20%|eCCA/GBC: uncommon	Ivosidenib	Phase III trial
*HER2* (*ERBB2*) overexpression/amplification *	eCCA, GBC (also seen in a subset of iCCA)	eCCA: ~17% HER2 overexpression|iCCA: ~5%|GBC: variable; higher than CCA in many series	Zanidatamab-hrii (HER2 bispecific antibody); trastuzumab-based regimens (context-dependent)	Phase II trials
*BRAF V600E* mutation	Pan-BTC (rare)	BTC overall: ~1–5% (reported in BTC; varies by cohort)	Dabrafenib + trametinib	Phase II trial
*NTRK* gene fusion	Tumor-agnostic (very rare in BTC)	BTC/GBC/CCA: <1%	Larotrectinib, entrectinib, repotrectinib	Phase II basket trials
MSI-H/dMMR	Tumor-agnostic (rare)	BTC overall MSI-H: ~1–3% (range across studies)	Pembrolizumab	Phase II trials

* For *HER2*, studies variably report overexpression by immunohistochemistry (IHC) vs. amplification by in situ hybridization (ISH) or next-generation sequencing (NGS); prevalence depends strongly on assay and positivity threshold. Reported alteration frequencies vary by assay platform (IHC/ISH/NGS), positivity definitions, and cohort composition; ranges above reflect commonly cited estimates stratified by anatomic subtype where available. Cross-study prevalence comparisons should therefore be interpreted cautiously.

**Table 2:** Selected approved and investigational fibroblast growth factor receptor (FGFR) inhibitors in *FGFR2*-rearranged biliary tract cancer.

Agent	Type/Selectivity	Mechanism of Inhibition	Key Clinical Trials	Population	Outcomes *	Regulatory Status in US	Reference
Pemigatinib	FGFR1-3 selective	Reversible ATP-competitive	FIGHT-202 [NCT02924376]	Previously treated CCA	ORR: 37.0% mPFS: 7.0 mo mOS: 17.5 mo	FDA accelerated approval in 2020	[15,38]
Infigratinib	FGFR1-3 selective	Reversible ATP-competitive	NCT02150967	Previously treated CCA with *FGFR2* fusion/rearrangement	ORR: 23.1% mPFS: 7.3 mo mOS: 12.2 mo	FDA accelerated approval in 2021, withdrawal in 2024	[34]
Derazantinib	FGFR1-3, multikinase	Reversible ATP-competitive	FIDES-01 [NCT03230318]	Previously treated iCCA with *FGFR2* fusion/rearrangement	ORR: 21.4% mPFS: 7.8 mo mOS: 15.5 mo	No further development	[27,39]
Futibatinib	FGFR1-4 pan-selective	Irreversible (covalent)	FOENIX-CCA2 [NCT02052778]	Previously treated iCCA with *FGFR2* fusion/rearrangement	ORR: 42% mPFS: 9.0 mo mOS: 21.7 mo	FDA accelerated approval in 2022	[16,40]
Erdafitinib	FGFR1-4 pan-selective	Reversible ATP-competitive	RAGNAR [NCT04083976]	Previously treated *FGFR*-altered non-urothelial solid tumors	In CCA ORR: 60.0% mPFS: 8.4 mo mOS: 18.7 mo		[41]

* Efficacy outcomes are reported from separate single-arm phase I/II studies and basket trial subsets. Cross-trial comparisons, including contrasts with historical chemotherapy outcomes, are exploratory only and limited by differences in patient populations, molecular selection, prior therapies, and study design. These data should not be interpreted as head-to-head comparisons.

**Table 3:** Summary of on-target and off-target resistance mechanisms to fibroblast growth factor receptor (FGFR) inhibitors in *FGFR2* fusion-positive iCCA.

Resistance Category	Representative Alterations	Effect on Drug Sensitivity	Therapeutic Implications
On-target *FGFR2* kinase-domain mutations	*N550D/K/H, E566A, V565F/L/I, K659M, C492R*	Reduced binding of reversible TKIs; partial retention with irreversible futibatinib for some variants	Switch from reversible to irreversible FGFR inhibitor; consider trial of next-generation allele-specific agent
Polyclonal *FGFR2* mutations	Multiple distinct *FGFR2* mutations in same patient	Co-existence of sensitive and resistant clones	Combination therapy or next-generation TKI with broader coverage
Bypass signaling activation	*KRAS/NRAS/BRAF* mutations, *MET/EGFR/ERBB2* amplifications	Reactivation of MAPK pathway independent of FGFR2	Combine FGFR and MAPK/receptor tyrosine kinase (RTK) inhibitors in trial setting
Phenotypic transition (EMT)	Upregulation of *TWIST1, SNAIL, ZEB1*; loss of E-cadherin	Decreased FGFR dependency	Investigational epigenetic or EMT-targeting strategies
Histologic transformation/clonal evolution	Acquisition of non-epithelial features	Resistance to FGFR blockade and chemotherapy	Re-biopsy to guide treatment choice

## Data Availability

No new data were created or analyzed in this study. Data sharing is not applicable to this article.

## Abbreviations

The following abbreviations are used in this manuscript:

BTC	Biliary tract cancer
iCCA	Intrahepatic cholangiocarcinoma
OS	Overall survival
mOS	Median overall survival
ICI	Immune checkpoint inhibitor
FGFR	Fibroblast growth factor receptor
IDH1	isocitrate dehydrogenase 1
BRAF	B-Raf proto-oncogene
HER2	human epidermal growth factor receptor 2
FGF	Fibroblast growth factor
RAS	Rat sarcoma
MAPK	mitogen-activated protein kinase
PI3K	phosphoinositide 3-kinase
AKT	protein kinase B
STAT	signal transducer and activator of transcription
FRS2	fibroblast growth factor receptor substrate 2
GRB2	growth factor receptor-bound protein 2
RAF	rapidly accelerated fibrosarcoma kinase
MEK	mitogen-activated protein kinase
ERK	extracellular signal-regulated kinase
mTOR	mechanistic target of rapamycin
PLCγ	phospholipase C γ
JAK	Janus kinase
eCCA	Extrahepatic cholangiocarcinoma
GBC	Gallbladder cancer
NTRK	neurotrophic tyrosine receptor kinase
ALK	anaplastic lymphoma kinase
ROS1	c-ros oncogene 1
ERBB2	erb-b2 receptor tyrosine kinase 2
IHC	Immunohistochemistry
ISH	In situ hybridization
NGS	Next-generation sequencing
TME	Tumor microenvironment
EGFR	epidermal growth factor receptor
EMT	Epithelial–mesenchymal transition
ORR	Objective response rate
PFS	Progression-free survival
mPFS	Median progression-free survival
TKI	Tyrosine kinase inhibitor
CCA	Cholangiocarcinoma
DOR	Duration of response
TEAE	Treatment emergent adverse event
mo	Month
FDA	The U.S. Food and Drug Administration
ctDNA	Circulating tumor DNA
RTK	Receptor tyrosine kinase
PROTAC	Proteolysis-targeting chimera
ADC	Antibody-drug conjugate
